# California State Veterinary Medical Association

**Published:** 1895-01

**Authors:** R. A. Archibald

**Affiliations:** Secretary


### CALIFORNIA STATE VETERINARY MEDICAL ASSOCIATION.

The California State Veterinary Medical Association held its annual meeting at the Baldwin Hotel, San Francisco, Cal., December 12, 1894.

The meeting was called to order by the President, Dr. H. A. Spencer. Upon roll-call the following-named gentlemen answered to their names: Drs. Maclay, Spencer, Sr., Wadams, Orvis, Fox, Archibald, Pierce, Jackson, Robin, Fabbi, Forrest, Williams, Graham, Eddy, O'Rourke, and Hogarty.

The minutes of the previous meeting were read and approved.

The annual reports of the Secretary and Treasurer were received and placed on file.

The Board of Examiners were granted further time on the application of Dr. J. A. Edmons.

The Legislative Committee, through their chairman, submitted the following report: Your Committee on Legislation beg leave to submit the accompanying bill for your consideration and adoption; further, we would recommend the adoption of the following resolution:

*Resolved*, That the bill as reported be presented at the next session of the Legislature, and that said committee be and they are hereby empowered to watch the progress of said bill through the Legislature. All of which is respectfully submitted.

The Secretary read the bill, whereupon, on motion of Dr. Fox, the report was adopted.[1]

The Secretary moved that five hundred copies of the bill be printed in circular form and distributed among the members of the Association. The motion was duly seconded and carried.

The Committee on Clinical Entertainment reported that they had procured several cases for the entertainment. Upon motion the report was accepted and the committee granted further time.

Under the head of applications for membership, the Secretary submitted the following names: J. R. Shaw, A. B. Wise, J. A. Edmons, J. Trullinger, J. J. Streets, B. W. Schodde, J. W. O'Rourke, S. A. Withers, A. J. Withers, E. J. Creely, and O. C. Baldy. The names were referred to the Board of Examiners.

The election of officers for the ensuing year resulted as follows: *President*—Dr. C. B. Orvis, of Stockton; *Vice-President*—Dr. R. T. Whittlesey, of Los Angeles; *Secretary*—Dr. R. A. Archibald, of Sacramento; *Treasurer*—Dr. D. F. Fox, of Sacramento; *Board of Examiners*—Drs. Maclay, Egan, Spencer, Sr., Graham, and Lemke; *Board of Directors*, the several officers of the Association.

The Secretary presented a bill for $10.50 for Secretary's supplies, etc., which was ordered paid.

The Secretary moved that when this Association adjourns it adjourns to meet in San Jose. The motion was duly seconded and carried.

Dr. Fox suggested the advisability of the Association indorsing some one of its members for the position of State Veterinarian. The matter was discussed in an informal manner by Drs. Spencer, Orvis, Fox, Pierce, and the Secretary. During the discussion Dr. Maclay moved to adjourn for dinner. On the motion being put the Chair, not being satisfied with an aye and no vote, called for a rising vote, which resulted in a tie, whereupon the Chair cast the deciding vote in favor of the motion. The meeting then adjourned to meet at 7 P.M.

*Evening Session*. At 7 P.M. the meeting was called to order by the President, Dr. H. A. Spencer, who called upon the President-elect to take the chair.

The new President, Dr. C. B. Orvis, upon taking the chair, delivered a few appropriate and well-chosen remarks, and as he was going to act in the capacity of an entertainer, he requested Dr. Spencer to take the chair for the rest of the evening.

Under the head of reading of papers, etc., and discussions, the Chair called upon Dr. C. B. Orvis to entertain the meeting, which he did by reporting three cases of intestinal obstruction from the formation of tumors and abscesses in the sublumbar region.

The reports were very interesting and instructive, and a very animated discussion followed their reading. The Chair closed the discussion with a few appropriate remarks.

[1] Synopsis of bill printed in this number.

Dr. W. E. Wadams was then called upon to entertain the meeting, which he did by offering an instructive and exhaustive paper on "Canine Distemper." The essayist described the disease in all its forms, giving the different symptoms and the different methods of combating the same. He also thoroughly described the different complications that might arise.

The reading of the paper was followed by a lively discussion, which was participated in by Drs. Maclay, Archibald, Orvis, Eddy, Fox, Pierce, Fabbi, O'Rourke, and Jackson. The Chair closed the discussion with a few remarks, and then called for reports of peculiar cases.

The Secretary reported a case of a cow whose milk contained so much acid that it was for all practical purposes useless. He said the cow had been changed from one food to another without changing the character of the milk. He also reported a few cases of what he supposed to be chronic pharyngitis, and he said that in some localities the disease seemed to be very prevalent in the late summer and fall of the year.

Dr. Orvis reported some cases of actinomycosis, and he exhibited some specimens taken from the tongues of two cows showing the ravages of the disease. He said the cows were almost starved to death when he was called in to see them.

Under the head of new business, Dr. Maclay offered the following resolutions:

*Whereas*, It has pleased the great Architect of the Universe to call from our midst one of our respected members; therefore, be it

*Resolved*, That in the death of Peter Burns this Association loses one of its most active members and most ardent supporters; as a man, as a citizen, as a husband, and as a father, he was beloved by all.

*Resolved*, That the heartfelt sympathy of the members of this Association be and it is hereby extended to the family of the deceased in this their hour of bereavement.

*Resolved*, That a copy of these resolutions be spread upon the minutes and a copy forwarded to the family.

Upon motion the resolution was adopted unanimously.

Dr. Maclay read the following resolution:

*Resolved*, That the thanks of this Association be and are hereby extended Dr. H. A. Spencer for the faithful and impartial manner in which he has performed the arduous duties of the office of President during the past year.

Upon motion the resolution was adopted.

The Secretary offered the following resolution:

*Whereas*, It has come to our notice that there are some of the members of this Association, and some who intend becoming members, who are violating Section 1 of the Code of Ethics, which states "that no member shall assume a title to which he has not a just claim;" therefore, be it

*Resolved*, That it is the sense of this Association that any member who refuses to discard, or who in violation of the Code of Ethics adopts, a title to which he has not a just claim be expelled from the Association, and have his name stricken from the roll of membership.

Considerable discussion followed, but finally, on motion of Dr. Maclay, the motion was adopted.

The Chair appointed the following-named gentlemen as essayists for the next meeting: Drs. Spencer, Sr., Faulkner, and Jackson.

On motion by the Secretary, a vote of thanks was extended to the essayists for the able and masterly manner in which they had entertained the meeting.

There being no further business to come before the meeting, it adjourned to meet in San Jose on Wednesday, March 13, 1895.

At 9 A.M. on Thursday, December 13, 1894, the members assembled at Dr. J. W. O'Rourke's hospital, at 331 Golden Gate Avenue, San Francisco, to witness a clinical entertainment. The first case was the extirpation of an actinomycotic tumor from the sublingual region in a cow. The operation was skilfully performed by Drs. Spencer and Fox. The second operation was the removal of a melanotic tumor from the lumbar region of a horse by Dr. C. B. Orvis. The third operation on the programme was the removal of the ovaries from a bitch by Dr. F. E. Pierce. The fourth and last operation was the removal of the left arytenoid cartilage and the left vocal cords for the amelioration of a condition called "roaring" in a horse, by Dr. R. A. Archibald.

The entertainment was voted an entire success both as an instructive entertainment as well as an enjoyable one.

The members before adjournment, through the President, Dr. Orvis, at the suggestion of Dr. Fox, extended to Dr. O'Rourke a vote of thanks for the use of his hospital and for his kindness.

R. A. ARCHIBALD, V.S.,
Secretary.

